# Regional, Household and Individual Factors that Influence Soil Transmitted Helminth Reinfection Dynamics in Preschool Children from Rural Indigenous Panamá

**DOI:** 10.1371/journal.pntd.0002070

**Published:** 2013-02-21

**Authors:** Carli M. Halpenny, Claire Paller, Kristine G. Koski, Victoria E. Valdés, Marilyn E. Scott

**Affiliations:** 1 Institute of Parasitology and McGill School of Environment Macdonald Campus of McGill University, Ste-Anne de Bellevue, Quebec, Canada; 2 School of Dietetics and Human Nutrition Macdonald Campus of McGill University, Ste-Anne de Bellevue, Quebec, Canada; 3 Escuela de Nutrición y Dietética, Facultad de Medicina, Universidad de Panamá, Ciudad de Panamá, Panamá; London School of Hygiene & Tropical Medicine, United Kingdom

## Abstract

**Background:**

Few studies have investigated the relative influence of individual susceptibility versus household exposure factors versus regional clustering of infection on soil transmitted helminth (STH) transmission. The present study examined reinfection dynamics and spatial clustering of *Ascaris lumbricoides*, *Trichuris trichiura* and hookworm in an extremely impoverished indigenous setting in rural Panamá over a 16 month period that included two treatment and reinfection cycles in preschool children.

**Methodology/Principle Findings:**

Spatial cluster analyses were used to identify high prevalence clusters for each nematode. Multivariate models were then used (1) to identify factors that differentiated households within and outside the cluster, and (2) to examine the relative contribution of regional (presence in a high prevalence cluster), household (household density, asset-based household wealth, household crowding, maternal education) and individual (age, sex, pre-treatment eggs per gram (epg) feces, height-for-age, latrine use) factors on preschool child reinfection epgs for each STH. High prevalence spatial clusters were detected for *Trichuris* and hookworm but not for *Ascaris*. These clusters were characterized by low household density and low household wealth indices (HWI). Reinfection epg of both hookworm and *Ascaris* was positively associated with pre-treatment epg and was higher in stunted children. Additional individual (latrine use) as well as household variables (HWI, maternal education) entered the reinfection models for *Ascaris* but not for hookworm.

**Conclusions/Significance:**

Even within the context of extreme poverty in this remote rural setting, the distinct transmission patterns for hookworm, *Trichuris* and *Ascaris* highlight the need for multi-pronged intervention strategies. In addition to poverty reduction, improved sanitation and attention to chronic malnutrition will be key to reducing *Ascaris* and hookworm transmission.

## Author Summary

Control of soil transmitted helminth (STH) infections is of central importance to improving preschool child health because these infections can have long lasting consequences on growth and development. Our study in indigenous Ngäbe preschool children in western Panama was conducted over a period of 16 months. We monitored reinfection dynamics of three STH infections (*Ascaris*, *Trichuris* and hookworm) over two reinfection cycles to gain an understanding of regional, household and individual factors that influenced transmission of these infections among preschool children. Despite the rural setting, where virtually all households live under conditions of extreme poverty, we identified spatial clusters of high prevalence of *Trichuris* and hookworm in the most remote and poorest area, whereas *Ascaris* was present throughout the study area. Preschool children who were chronically malnourished (low height-for-age) had a higher reinfection burden of *Ascaris* and hookworm. Household poverty (low relative household wealth and maternal education) and infrequent latrine use were also influential in *Ascaris* reinfection. This cross-disciplinary analysis of preschool child STH transmission in a poor rural setting provides pertinent information for STH control programs that aim to break the cycle of poverty and infection.

## Introduction

The soil transmitted helminth (STH) infections, *Ascaris lumbricoides*, *Trichuris trichiura* and the hookworms *Ancylostoma duodenale* and *Necator americanus* are estimated to result in the loss of 39 million disability adjusted life years (DALYs) annually [1] and to have long-lasting implications for child physical and cognitive development [2]–[5]. To control the morbidity associated with these infections for high risk populations living in endemic communities, current public health programs focus on chemotherapy [6] through programs typically delivered to school children [7]. Although there is evidence from theoretical models [8] and community studies [9] that such efforts have spill-over effects that reduce transmission in the untreated portions of the population, ensuring control of infections in preschool children is particularly important given the impact of STH on early growth and development [5], [10].

Many studies have shown that the individuals most heavily infected prior to anthelmintic treatment have a high reinfection burden, and thus that some individuals are predisposed to heavy infection [11], [12]. Mechanisms used to account for this predisposition include genetic and nutritional components of susceptibility, behavioural patterns that directly promote contact with infective stages and factors that promote egg/larval survival in some domestic and peri-domestic environments. Susceptibility may be related to differences in the genetic regulation of B cell activation and immunoglobulin secretion [13], and to poor nutritional status [14]–[16] through the impaired immune function that accompanies micro and macronutrient deficiencies [10]. Behaviours including geophagy [17], [18], poor hygiene [19] and not wearing shoes [20] reportedly increase an individual's risk of contact with eggs or larvae. Household level factors associated with poverty [21], such as limited latrine access [16], [21], [22], within household crowding [23], [24] and low maternal education [21], [25], [26] increase environmental contamination with STH eggs and larvae and contribute to the patterns of household aggregation detected in community-level epidemiological studies [27], [28].

It has also been shown that STH infections cluster at the regional and national scales [29], [30]. Such clusters have been associated with biophysical and climatic features such as vegetation cover [31], [32], soil type [32], [33], rainfall, temperature and altitude [29], [31], [34] that influence egg and larval survival, and also with limited sanitation and hygiene infrastructure that promote transmission [35]. Given the challenge of disentangling the influence of individual, household and regional risk factors of STH transmission, spatial analysis is becoming more widely used in epidemiological studies. Three recent investigations included these methods and highlighted the relative importance of household-level socio-economic (SES) factors. In Uganda, a cross-sectional survey found that household variables influencing exposure play a greater role than host genetics in determining the distribution of hookworm infection intensity [36]. A longitudinal study assessing hookworm post treatment reinfection rates in Brazil found child sex and SES variables such as household construction were more influential than regional geographic variables such as rurality, altitude and soil moisture [37]. Another longitudinal study, in Bangladesh, found that household exposure risk factors accounted for more than half of the variability in household clustering of *Ascaris* infection and that after accounting for household clustering, individual predisposition to infection was minimal [38]. Thus it is well established that SES variables are influential in determining the spatial distribution and transmission of STH infections [36], [38]. However, few studies have the detailed individual data to investigate the relative influence of individual susceptibility due to chronic undernutrition versus household exposure variables on STH transmission within a context of regional clustering of infection in preschool children.

Our study was designed to examine the influence of spatial patterns of infection as well as household and individual factors on the transmission dynamics of *Ascaris lumbricoides*, hookworm, and *Trichuris trichiura* during two sequential reinfection cycles in preschool children in an impoverished, rural region of Panamá. Specifically, we aimed to: 1) compare reinfection dynamics among the three STH; 2) identify and characterize regional scale spatial clusters of high prevalence of infection; and 3) determine the relative contributions of individual factors (age, sex, height-for-age, pre-treatment intensity, latrine use), household factors (household density, asset-based household wealth index, household crowding, maternal education) and a regional factor (residence in a high prevalence cluster) on reinfection intensity of STH infections in preschool children.

## Materials and Methods

### Ethics Statement

Ethical approval was obtained from the Instituto Conmemorativo de Gorgas in Panamá and McGill University in Canada. The study was conducted in accordance with the Guía para Realizar Estudios e Investigaciones en los Pueblos Indígenas de Panamá, including initial and result-sharing workshops with participants in each village. Written informed consent was obtained from primary caregivers during a household visit that included an explanation of study significance and participant requirements and rights, as well as an opportunity to ask questions in Spanish and Ngäbere.

### Study Area

The comarca Ngäbe-Buglé is a semi-autonomous political region inhabited primarily by the Ngäbe and Buglé indigenous groups (2004 population estimate of 128,978) where over 90% of families live in extreme poverty (< US$1.75/day) [39]. In 2005 and 2007, two forms of the Conditional Transfer (CT) program Red de Oportunidades began in the comarca, providing an additional US$50/mo in either cash (2007) or food vouchers (2005) in exchange for participation in health and education programs. The research reported here is part of a larger study that was conducted in the district of Besiko in two adjacent corregimientos (Soloy and Emplanada de Chorcha), each accessible most of the year by one dirt road. As previously reported [40], household density varied considerably. More densely populated regions (>50 participant households/km^2^) were closer to a road and had better access to latrines, aqueducts and health facilities and had a higher average asset-based household wealth index (HWI).

### Study Design and Protocol

Our study was designed to estimate STH infection and reinfection in 2 treatment and reinfection cycles during a 16 month sample period (Cycle 1: 9 month reinfection period from July 2008 to April 2009; Cycle 2: 6 month reinfection period from April to October 2009). The study recruitment protocol has been described previously [40]. In brief, households with children from 0–48 mo of age and living in extreme poverty (defined as having participated in a CT program) from 12 randomly selected villages split evenly between the 2 corregimientos were invited to participate in the study. All but 3 of the 265 eligible households that were approached agreed to participate. Spatial data were unavailable for 12 households (missing or erroneous longitude and latitude coordinates). The household and demographic characteristics of the 12 households for which spatial data were unavailable did not differ from the other 250 participant households. Thus, the present analysis included a total of 250 households and 356 children (153 households with 1 eligible child, 88 with 2, and 9 households with 3 eligible children). Stool samples were collected at 7 household visits, and additional data from questionnaires and anthropometry were provided during 3 additional household visits. Temporary migration for agricultural purposes was common in the participant population and therefore few children were available at all household visits.

#### Cycle 1 stool samples and treatment

Baseline fecal samples for reinfection Cycle 1 were collected in June 2008 (n = 215), after which a single dose of Albendazole (ABZ) was distributed to all available participant children >12 mo of age (59%) according to Ministry of Health procedures (200 mg: 1–2 yrs, 400 mg: 3–5 yrs). These children were observed during treatment administration to ensure compliance. A post-treatment fecal sample was collected 2 wk later (n = 100) from which drug efficacy against *Ascaris* was assessed from the subsample of 16 children who had been infected and had received treatment. Reinfection was monitored through stool samples collected at 3 mo (n = 87) and 9 mo (n = 155) (Table 1) from children who had received treatment. From the 9 mo reinfection sample, 122 of the 155 children had also provided baseline samples for Cycle 1. At 9 mo, stool samples were also available from an additional 115 children who had not been treated in Cycle 1 either because they were too young or they were unavailable when the treatment was administered. These data have not been included in Cycle 1 but did form part of the baseline sample for Cycle 2.

**Table 1:** Sample sizes for two STH reinfection cycles among Panamanian preschool children.

	Baseline epg	Received Treatment	2 wk/3 wk epg	3 mo/4 mo reinfection epg	9 mo/6 mo reinfection epg
Cycle 1	215	209	100	87	155
Cycle 2	270	279	222	218	200

Reinfection epg sample sizes only include individuals who also received treatment. Cycle 1 Baseline, 2 wk and 3 mo epg estimates were calculated using Kato Katz methodology. Cycle 1 9 mo reinfection epg and all Cycle 2 epg estimates were calculated using FLOTAC.

#### Cycle 2 stool samples

The 9 mo reinfection sample (April 2009) also served as the baseline sample for Cycle 2 (n = 270), after which a single dose of ABZ was distributed as above to all available participant children >12 mo of age (78%) (Table 1). Three weeks later, drug efficacy was evaluated as above (*Ascaris* n = 32, hookworm n = 25, *Trichuris* n = 7) and those who remained infected with at least one STH (n = 18) were given a second dose of ABZ. Reinfection was monitored through stool samples collected at 4 (n = 218) and 6 (n = 200) mo after ABZ treatment. In Cycle 2, 172 children provided baseline fecal samples, received treatment and provided a 6 mo reinfection sample. Forty eight percent of preschoolers enrolled in the study received treatment in both Cycle 1 and Cycle 2.

### Fecal Samples

Labelled collection containers and detailed instructions were given to each caregiver during household visits on the day prior to fecal sample collection. Samples were collected from the home the following morning and transported on ice to the Parasitology Laboratory at the Hospital General del Oriente, Chiriqui, Panamá.

The primary outcome in this study was intensity of infection, measured as epg. Hookworms were not identified to species level, however, *Necator americanus* is the predominant hookworm in Central America [41]. In Cycle 1, data on epg were obtained only from duplicate Kato Katz preparations [42] whereas in Cycle 2, both Kato Katz and the FLOTAC techniques [43] were used. For each nematode (*Ascaris, Trichuris* and hookworm), a comparison of the diagnostic ability (presence/absence) between FLOTAC and Kato Katz was conducted using Cohen's Kappa statistic on samples that were assayed using both techniques. Sensitivity of each method (expressed as a percentage) was also analyzed by dividing the number of positives for a given method by the total number of positives identified by either method. Finally, the correlation of intensity estimates between the two methods was examined using Spearman Rank correlation coefficients.

### Characterization of Infection Risk Factors

#### Individual factors

Age and sex were recorded from child health cards during household interviews. Child latrine use (n: 2008 = 279, 2009 = 286) was obtained from questionnaires. Pre-treatment epgs were measured as above, and were used both as an indicator of baseline infection status, and also as an indicator of individual predisposition to reinfection [44], [45]. Based on previous observations that child stunting was associated with more rapid reinfection in a nearby population [46], height-for-age Z score (HAZ) was used to assess individual susceptibility to STH reinfection. Height/length of participating children was measured by trained nutritionists using Portable Stadiometers (Seca 214, Birmingham, UK) and Measuring Mats (Seca 210, Birmingham, UK). Child HAZ scores (2008: n =  285, 2009: n = 264) were calculated from WHO growth reference standards using WHO Anthro 3.1 [47]. Children were classified as stunted if their HAZ was < **−**2SD.

#### Household factors

The primary caregivers of participating children were interviewed in Spanish or Ngäbere using field tested questionnaires during several household visits to gather information related to factors that directly influence exposure to infection. Years of school attended by the mother (n = 240), and number of family members per room (measure of household crowding; n = 217) were recorded. Information on 11 variables relating to household construction (dirt floor, absence of walls, solid walls), possessions (radio, cell phone, bicycle, sewing machine, stove, hoe) and access to running water and latrine (n = 229) were used to calculate an asset-based Household Wealth Index (HWI) that describes relative poverty within our population as previously described [40]. Geographic coordinates recorded with a handheld Geographic Positioning System (GPS) (Garmin eTrex Vista HCX, Olathe KS) (n = 250) were used to characterize the spatial dispersion of participant homes [40]. Resulting density estimates for each household described the number of other study participant homes found within a radius of 250 m, expressed in homes per square kilometer.

#### Regional factor

We also conducted spatial analyses across the study area on the location of households with an infected preschool child at baseline of both Cycle 1 and Cycle 2. Spatial clusters of “infected” households were detected using SaTScan software (Boston, MA). Kulldorf's Space-Time Scan Statistic uses a scanning circular window to identify distinct clusters of infection. For each location the circular window varied in size from zero to 50% of the population and the distribution of cases inside the window was compared to that outside the window. Likelihood ratios based on a Bernoulli probability model identified high prevalence clusters as those where the number of households with at least one infected preschool child was greater than expected if the infection was randomly dispersed among households (p<0.05) [48], [49]. This approach revealed whether or not regional clusters were present for each STH at baseline of each Cycle, and if present, where they were located.

### Statistical Analysis

All statistical comparisons were conducted using STATA 11.1 (College Station, TX). In all cases, the level of significance was set at p<0.05. Binomial confidence limits (95%) for prevalence data were determined using the Agresti-Coull calculation and comparisons were conducted using contingency tables and *X* ^2^ tests. Continuous data were reported as the mean ± SEM, unless otherwise stated. Univariate comparisons between households within and outside high prevalence clusters, as well as between infected and uninfected individuals at both the 9 mo (Cycle 1) and 6 mo (Cycle 2) reinfection sample periods, were conducted using non-parametric Mann-Whitney, Kruskal-Wallis and Spearman correlation analyses due to the non-normal distribution of the data (e.g. maternal education, HWI, age, epg). Univariate analyses related to reinfection included only those children who had provided a 9 mo/6 mo reinfection sample and who had received treatment (Cycle 1, n = 155; Cycle 2, n = 200).

Egg Reduction Rates (ERR) were calculated as the mean percentage reduction in epg [50], using epgs from the Kato Katz for Cycle 1 and from FLOTAC for Cycle 2.

Step-wise logistic regression models were used to examine which household risk factors were associated with presence in the high prevalence clusters detected by the SaTScan software (hookworm and *Trichuris* at baseline of Cycle 2). Step-wise negative binomial regression models were also performed to determine the impact of three sets of risk factors on reinfection epgs: 1) regional factor, namely residence in a high infection cluster; 2) household factors, namely HWI, household density, household crowding, maternal education); and 3) individual factors, namely age, sex, HAZ, predisposition measured by pre-treatment epg, latrine use). These analyses were done for *Ascaris* reinfection in both Cycle 1 and Cycle 2 and for hookworm only in Cycle 2 when the more reliable FLOTAC epgs were available. No regression models were developed for *Trichuris* reinfection because of the low drug efficacy. Final models included variables with p<0.10. The Huber estimator for robust standard error estimation was used to account for clustering at the household level. Multivariate analyses were limited to individuals who had received treatment and provided baseline and 9 mo or 6 mo reinfection samples and for whom a complete set of data on risk factors were available (Cycle 1, n = 100; Cycle 2, n = 140).

## Results

### Methodological Comparison

Although there was significant concordance between the methods for detection of all three parasites (*Ascaris*: k = 0.91, p<0.001; *Trichuris*: k = 0.66, p<0.001; hookworm: k = 0.52, p<0.001; n = 604), FLOTAC was more sensitive than Kato Katz in the detection of hookworm (93% vs 45% of known positive samples, respectively) and *Trichuris* (97% vs 56% of known positive samples, respectively). Spearman rank correlation analysis of intensity estimates among samples identified as positive by both methods (n = 196) revealed a positive correlation between methods for *Ascaris* epg (r = 0.79, p<0.001), hookworm (r = 0.57, p<0.001) and *Trichuris* (r = 0.51, p = 0.003). Comparison of the mean intensity of infection revealed that estimates of *Ascaris* intensity measured by Kato Katz were higher than those measured using FLOTAC (Kato Katz: 18393±1906; FLOTAC: 5364±1017; p<0.001) but mean intensity estimates for hookworm and *Trichuris* did not differ by method (Hookworm: Kato Katz: 3601±826; FLOTAC: 385±72; p = 0.16; *Trichuris*: Kato Katz: 3357±1249; FLOTAC: 227±32; p = 0.39). Due to the greater sensitivity of FLOTAC to hookworm and *Trichuris* infection, the similar diagnostic ability of both methods for *Ascaris* and the larger number of samples examined by FLOTAC in 2009, analysis of infection prevalence and intensity used FLOTAC estimates whenever available.

### Household and Demographic Characteristics of Participants

Average density of participating households was 35±2 houses/km^2^ and the household wealth index (HWI) was 0.21±0.04. Latrines were available to 31% of households and piped water (aqueducts) to 34% of households. Households had an average of 5.4±0.2 people/room and mothers had 3.8±0.2 yrs of education. The average age of participating children at baseline of Cycle 1 was 31.4±0.9 months, 49% were female, and 72% were stunted. At the beginning of Cycle 2 the average child age was 36.9±0.9 months, 49% were female and 69% were stunted. Child demographic data presented here relates to the subset of children who received treatment in each Cycle, which differ slightly, but not significantly, from the total population reported elsewhere [40].

### Reinfection Dynamics

Although our first objective had been to compare infection and reinfection dynamics among the three STH infections over 2 consecutive reinfection cycles, the absence of FLOTAC data during Cycle 1 limited our analysis only to *Ascaris*.

**Figure 1:**
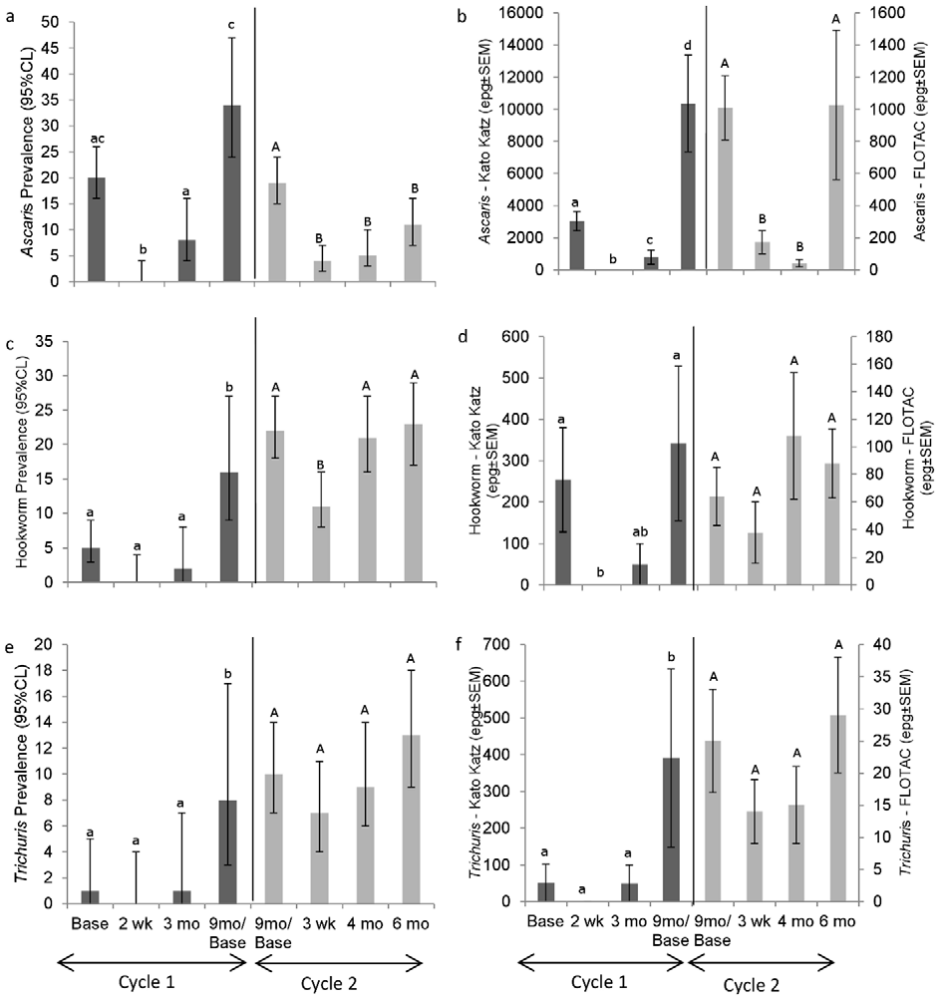
Prevalence and intensity for *Ascaris*, hookworm and *Trichuris* in Panamanian preschool children. Prevalence (A, C, E) and intensity (B, D, F) for *Ascaris* (A,B), hookworm (C,D) and *Trichuris* (E,F) were assessed by Kato Katz (dark grey bar – left vertical axis) and FLOTAC (light grey bar – right vertical axis) during two reinfection cycles. Different letters indicate significant differences (P<0.05) among sample periods (lower case Kato Katz comparisons; upper case FLOTAC comparisons). Solid vertical bar separates Kato Katz from FLOTAC data. A single dose of Albendazole (200 mg: 1–2 yrs, 400 mg: 3–5 yrs) was delivered after the baseline samples in each cycle and children who remained infected after treatment in Cycle 2 received a second dose. Data presented consider each cycle independently resulting in a different sample group for the 9 mo reinfection point of Cycle 1 and the baseline of Cycle 2. Each data point represents the population estimates based on all available fecal samples at each time point for children who received treatment at baseline of the respective cycle.

*Ascaris* was detected in 20% of children prior to ABZ treatment in Cycle 1 (Figure 1 A) with relatively low mean intensity (Figure 1 B). Treatment with ABZ had an *Ascaris* ERR of 100% in the subsample for whom drug efficacy was assessed. Within 3 mo the prevalence of *Ascaris* in those who had received treatment had increased to 8%, half that recorded at baseline; by 9 mo, 34% of the children were infected and intensity of reinfection was three times higher than baseline values. At the baseline of Cycle 2, 19% of all individuals who provided samples were infected. ABZ efficacy against *Ascaris* was 97%. Despite the low prevalence of *Ascaris* at the end of Cycle 2 (11%), the intensity had increased to pre-treatment levels (Figure 1 B).

Hookworm prevalence was 5% at the baseline of Cycle 1 (Figure 1 C), with a low average intensity (Figure 1 D). Drug treatment with ABZ eliminated infection in the 3 infected children. By 3 mo post treatment, hookworm prevalence (Figure 1 C) and intensity (Figure 1 D) were similar to baseline levels and by 9 mo post treatment both metrics had exceeded baseline levels. In Cycle 2, ERR was 89% and both prevalence and intensity had returned to pre-treatment levels within 4 mo of treatment.

*Trichuris* prevalence was only 1% at the baseline of Cycle 1 (Figure 1 E) and therefore it was not possible to calculate ERR. Three months after treatment, the prevalence remained very low, but by 9 mo post treatment, *Trichuris* prevalence (Figure 1 E) and intensity (Figure 1 F) reached the highest values detected during the study. A single dose of ABZ at the beginning of Cycle 2 led to only a 40% ERR for *Trichuris*. The 4 and 6 mo post-treatment prevalence and intensity for *Trichuris* did not differ from Cycle 2 baseline values.

### Factors Associated with Spatial Clusters of Infection and with Individual Reinfection Intensity

#### Hookworm

**Figure 2:**
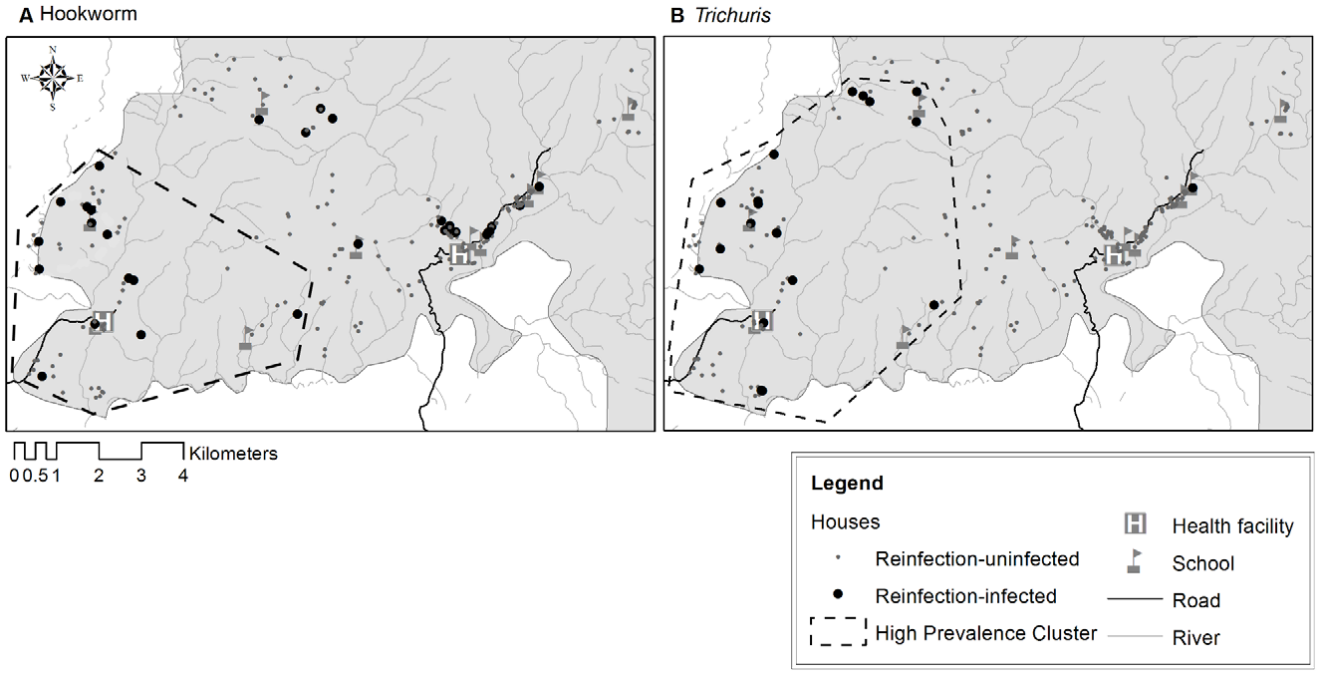
Spatial clusters of households with high prevalence of hookworm and *Trichuris* infection. High prevalence clusters (dotted line) for hookworm (A) and *Trichuris* (B) infection detected at Baseline of Cycle 2. Uninfected (small grey dots) and infected households (large dark dots) based on data from Cycle 2 at 6 mo (hookworm and *Trichuris*).

A region of high prevalence incorporating 23% of households (Log likelihood ratio 9.97 p = 0.01) was detected at baseline of Cycle 2 (Figure 2 A). At 6 months post treatment in Cycle 2, 41% of the homes in the high prevalence cluster had an infected child. The high prevalence cluster was characterized by lower HWI, lower household density, less access to physical infrastructure and lower maternal education compared with households outside the cluster (Table 2). Multivariate models confirmed that low household density, was a risk factor for residing in a high infection cluster (Table 3).

**Table 2:** Comparison of characteristics between households within and outside the high prevalence clusters^1^.

	Hookworm		*Trichuris*	
	In (n = 58)	Out (n = 195)	In (n = 63)	Out (n = 190)
Household Factors				
Household Density, *houses/km* 2	13±1	41±3**	13±1	42±3**
Wealth Index, *HWI* 2	−0.04±0.06	0.27±0.05*	−0.08±0.06	0.30±0.05*
Latrine Access, %	18(10–30)	35 (28–42)*	16(9–27)	36(29–43)*
Aqueduct Access, %	16(8–27)	40(33–47)*	14(7–25)	41(34–48)**
Mother's Education, *yrs*	2.9±0.4	4.1±0.3*	2.8±0.4	4.2±0.3*
# People/Room	4.7±0.3	5.6±0.2	5.2±0.3	5.4±0.2

1 Summary statistics presented are mean ±SEM or % (95% CL).

2 Asset based index, weights derived from the first component of Principle Components Analysis.

* p<0.05;

** p<0.001.

**Table 3:** Final multiple logistic regression models predicting household presence within Cycle 2 high prevalence clusters^1^^,^ 2.

	Hookworm	*Trichuris*
Household Factors		
Household Density, *houses/km* 2	0.95 (0.93–0.97)	0.96 (0.93–0.98)
Wealth Index, *HWI* 3	NE^4^	0.53 (0.28–0.98)
Model Statistics		
n	231	231
*X* 2	38.84	47.40
P	<0.001	<0.001

1 Odds ratios ±95% CI.

2 Latrine access and maternal education did not enter any model.

3 Asset based index, weights derived from the first component of Principle Components Analysis.

4 NE = Variable was excluded during the stepwise process (p>0.10).

At the individual child level, univariate analysis revealed that those who were infected at the end of Cycle 2 were more likely to live in a high prevalence cluster and had heavier baseline infection burdens than uninfected children (Table 4). Spearman rank correlation confirmed the correlation between baseline and reinfection epgs (r = 0.35, p<0.001). After multivariate analysis, at the end of Cycle 2, only individual level factors influenced reinfection epg. Children who had a low HAZ score were most heavily infected and might have had a higher pre treatment epg (p = 0.06) (Table 5).

**Table 4:** Comparison of regional, household and individual factors influencing STH reinfection of Panamanian preschool children^1^.

	Cycle 1			Cycle 2				
		*Ascaris*			*Ascaris*		Hookworm	
	n	Infected	Uninfected	n	Infected	Uninfected	Infected	Uninfected
Regional Factors								
Residence in high prevalence cluster, %		-	-	144			41(25–58)	20(14–29)*
Household Factors								
Household Density, *km* 2	131	45±9	31±3	144	31±9	35±3	39±7	33±3
Wealth Index, *HWI* 2	128	0.17±0.13	0.20±0.07	140	−0.06±0.16	0.16±0.06	0.06±0.1	0.16±0.06
Mother's Education, *yrs*	131	3.1±0.7	3.5±0.3	143	2.1±0.8	3.7±0.33	3.5±0.7	3.5±0.3
# People/Room	123	5.3±0.6	5.2±0.3	143	5.5±0.6	5.5±0.2	5.8±0.5	5.5±0.3
Individual Factors								
Cycle 1 Baseline Infection, *epg*	122	10463±3119	1737±478**	172	2895±1549	612±193*	91±39	4±2**
Age, *mo*	155	32±2	31±1	200	22±3	27±1	29±2	25±1
Female, *%*	155	48(33–65)	52(44–61)	200	45(27–65)	52(45–59)	47(33–61)	53(45–61)
Height for age								
Z score	130	−2.8±0.2	−2.6±0.1	170	−2.6±0.2	−2.4±0.1	−2.6±0.2	−2.4±0.08
Stunting, *%*	130	84(67–93)	70(60–78)	170	78(54–92)	70(63–77)	77(61–88)	70(61–77)
Latrine use, *%*	135	9(2–24)	18(11–26)	185	10(2–31)	15(10–21)	12(5–26)	15(10–22)

1 Summary statistics presented are mean ±SEM or % (95% CL).

2 Asset based index, weights derived from the first component of Principle Components Analysis.

* p<0.05;

** p<0.001.

**Table 5:** Negative binomial regression models of *Ascaris* and hookworm reinfection intensity in Panamanian preschool children.

	*Ascaris*	*Ascaris*	Hookworm
	Cycle 1	Cycle 2	Cycle 2
	9 mo	6 mo	6 mo
	IRR^1^	IRR	IRR
Regional Factors			
In High Cluster, baseline Cycle 2^2^	NA^3^	NA	NE
Household Factors			
Household density, *homes/km* 2	NE^4^	0.96 (0.93–1.001)	NE
Household wealth, *HWI*	0.19 (0.08–0.48)**	145 (7–3012)*	NE
Mother's education, *yrs*	NE	0.48 (0.28–0.80)*	NE
Individual Factors			
Age, *mo*	NE	0.93 (0.88–0.99)*	NE
Sex^5^	NE	27 (2–303)*	NE
Baseline Infection, *epg*	1.0001 (1.0000–1.0002)**	1.0003 (1.0001–1.0006)*	1.004 (1.00–1.009)
Height-for-Age, *Z score*	0.15 (0.07–0.32)**	NE	0.49 (0.29–0.84)*
Latrine use^6^	0.007 (0.001–0.04)**	0.0001 (0–0.05)*	NE
Model Statistics			
N	100	140	140
P	<0.001	<0.001	<0.001
Wald chi^2^	104.74	55.95	10.49
1/Alpha^7^	0.04(0.02–0.06)	0.01 (0.007–0.02)	0.02 (0.01–0.04)

1 Incidence Rate Ratio.

2 Household within identified high prevalence cluster of infection by SaTScan Spatial Scan (0 = no, 1 = yes).

3 NA = Not applicable.

4 NE = Variable was excluded during the stepwise process (p>0.10).

5 0 = boy, 1 = girl.

6 0 = no, 1 = yes.

7 1/Alpha statistic indicates the degree of overdispersion in the data.

* p<0.05,

** p<0.001.

Variables that did not enter any model: Household crowding.

#### *Trichuris trichiura*

As with hookworm, a cluster of high *Trichuris* prevalence (25% of participant households: Log likelihood ratio = 11.8928.40 p = 0.002) was detected at baseline of Cycle 2 (Figure 2 B). At 6 months post treatment 38% of households in the high prevalence cluster had an infected child. The cluster of high infection prevalence was characterized by lower household density, lower HWI, less access to physical infrastructure and lower maternal education than households outside the high prevalence cluster (Table 2). Multivariate analysis confirmed that the high prevalence cluster was less densely populated and households had a lower HWI (Table 3).

Given the low ABZ efficacy of a single dose of ABZ against *Trichuris*, and given that only 18 children received a second dose of ABZ, the 4 and 6 mo prevalence and intensity values in Cycle 2 likely reflect continuing *Trichuris* infection rather than rapid reinfection. Therefore univariate and multivariate analysis of individual reinfection in Cycle 2 was not conducted.

#### *Ascaris lumbricoides*

No high prevalence clusters were identified for *Ascaris* infection, indicating the widespread occurrence of infection throughout the region. At the individual child level, univariate comparison between infected and uninfected preschool children at the end of both Cycle 1 and Cycle 2 showed that infected preschool children had higher pre-treatment infection burdens (Table 4). Consistent with this, there were positive correlation between baseline and reinfection epgs (Cycle 1: r = 0.35, p<0.001; Cycle 2: r = 0.24, p = 0.002). No other differences were detected in the univariate analyses.

Composite models emerging from the multivariate analysis of Cycle 1 reinfection epg confirmed that pre-treatment infection burden was a risk factor for higher reinfection intensity and identified two additional individual level factors. Low HAZ was associated with a higher reinfection egp whereas latrine use was associated with a lower reinfection epg. Among the household factors, higher HWI was associated with lower *Ascaris* reinfection (Table 5). In reinfection Cycle 2, individual factors (higher pre-treatment epg, younger age, female, not using a latrine) were associated with increased reinfection epg. At the household level, reinfection epg was higher in households where mothers had less education, and surprisingly, in households with a greater HWI (Table 5).

## Discussion

Our study builds on past work in this rural indigenous area of Panamá and further characterizes the epidemiology of STH infections. As shown by previous empirical studies [51], [52], theoretical studies [8] and a recent meta-analysis [12] of STH infection dynamics, we found that prevalence and intensity of infection returned to baseline levels following treatment. Our use of spatial analysis uncovered distinctions among STH infections. Whereas spatial clusters of *Trichuris* and hookworm were detected, and overlapped in a region characterized by poor development (as measured by low HWI and low household density) no spatial clustering was detected for *Ascaris*, indicating its more homogenous dispersion throughout the region. Our multivariate regression models revealed differences between *Ascaris* and hookworm in the relative impact of household and individual factors in driving reinfection dynamics. Neither regional nor household variables emerged in the model for hookworm, and after controlling for baseline epg, the only individual factor that emerged was HAZ. Reinfection models for *Ascaris* also included individual factors (baseline epg, HAZ, age, sex) in one or both reinfection cycles, as well as two household factors, maternal education and HWI. Interestingly, in Cycle 1, reinfection rates were higher in households with low HWI whereas the opposite pattern was seen in Cycle 2.

Our first objective was to compare reinfection dynamics among the three STHs. Due to differences in the sensitivity of FLOTAC and Kato Katz for hookworm and *Trichuris* infection intensity estimates, we have focused our comparison between reinfection cycles primarily on *Ascaris*, for which both diagnostic methods had similar sensitivity. For *Ascaris*, the reinfection profile was similar in both years, with epgs reaching pre-treatment levels within 6–9 mo. In contrast, our data indicate that reinfection with hookworm and *Trichuris*, measured as the change in epg, was more rapid during Cycle 1 between 3 and 9 mo post-treatment than during Cycle 2. Four factors may have contributed to this difference. First, Cycle 1 included the wet season (October – January) whereas Cycle 2 overlapped only briefly with the wet season. Indeed, more wet days [53] and soil wetness [54] are considered conducive to *Ascaris* transmission and hookworm larval abundance. Second, the reinfection period was longer for Cycle 1 (9 mo) than Cycle 2 (6 mo). Third, the Conditional Transfer programs in the region had incorporated anthelmintic delivery through school based programs for at least 1 year prior to this study. A slower rate of reinfection in subsequent treatment cycles is characteristic of an area with effective control [55], [56] even in the segments of the population not targeted by treatment programs [6], [9]. Fourth, at least in the case of *Trichuris*, the low efficacy of ABZ precludes us from assuming that the 6 mo epg at the end of Cycle 2 is driven by reinfection. Over time, epgs reach an equilibrium determined by the Basic Reproduction Ratio of the parasite and the host and environmental characteristics of the region thus *Trichuris* epgs after ABZ treatment may have been close to this equilibrium. Of further note is that the three STH species did not demonstrate the expected reinfection dynamics. Based on the longevity of the STH species, *Ascaris* and *Trichuris* are expected to reach baseline intensity more quickly than hookworm [51] however in our study, hookworm reinfection intensity reached baseline levels in 4 mo compared to the 6 mo that it took for *Ascaris* and *Trichuris* infections to reach pre-treatment intensity. We suggest that the lower treatment efficacy of a single dose of ABZ for hookworm (ERR = 89%) compared to *Ascaris* (ERR = 97%) and the higher baseline prevalence of hookworm infection (21%) compared to *Trichuris* (10%) led to increased transmission of hookworm by increasing the number of hookworm infectious stages in the environment relative to *Ascaris* and hookworm and thus increasing the efficiency of transmission [55].

The low efficacy of ABZ against *Trichuris* has been noted in other studies [57]–[60] and, in this *Trichuris* population, may be related to the genetic polymorphisms associated with benzimidazole resistance that have been detected in *Trichuris* eggs from our study area [61]. ABZ may not be the most appropriate drug to use for *Trichuris* in this region, and its continued use, even if directed towards control of *Ascaris* and hookworm could lead to an increase in the frequency of these polymorphisms and greater evolutionary pressure toward ABZ resistance in *Trichuris*.

Our second objective was to identify and characterize spatial clusters of high STH prevalence. Two intriguing observations emerged. First, the location of hookworm and *Trichuris* high prevalence clusters significantly overlapped and the clusters shared the common characteristic of low household density. In our study area, we have previously reported that low household density was associated with lower HWI, as well as being farther from roads and health centres [40], and thus we have considered low household density as an indicator of poor regional development or “remoteness”. The relationship between “remoteness” and clusters of high prevalence of infection could also be linked to characteristics of the physical environment [32], [33], to high risk activities that occur in these areas [21], [62], and to the lack of sanitation and hygiene infrastructure [40]. Surprisingly, residence in a high prevalence cluster did not emerge as a risk factor for hookworm reinfection intensity. This could be explained because the greater isolation of houses in the high prevalence cluster might reduce contact of children with hookworm larvae in neighbouring homes, compared with more densely spaced homes. Alternatively, by controlling for baseline epg, we may have reduced the influence of the regional scale clustering of infection. Second, no high prevalence clusters of *Ascaris* were detected. *Ascaris* was the most prevalent parasite in our study area. The long survival and the stickiness of *Ascaris* eggs [51] may facilitate their dispersion through the environment leading to more evenly distributed egg exposure, and may explain the generalized infection throughout the regions.

Our final objective was to compare the relative contribution of individual, household and regional factors in the transmission dynamics of *Ascaris* and hookworm. Predisposition to heavy infection is characteristic of STH infection and has been noted throughout the developing world [44], [45], [63], [64]. Our multiple regression analyses on epg of individual children further supported these findings as pre-treatment epg was a strong predictor of reinfection intensity for *Ascaris* and may have also influenced hookworm reinfection epg. Mechanisms to explain predisposition include the influence of individual traits on susceptibility such as genetic differences in immunity [65] or nutritional status [10], as well as household factors that increase exposure to infection [38].

After controlling for baseline epg, HAZ emerged as an individual factor in models of reinfection intensity for hookworm (Cycle 2) and *Ascaris* (Cycle 1). Most longitudinal studies examining STH infection and anthropometric outcomes have focused on the potential benefit of anthelmintic treatment on weight or height gain [66], [67] rather than the impact of undernutrition on child susceptibility to infection or reinfection. The few studies that have specifically examined the latter relationship have either not controlled for potential confounding factors [14] or have found that the increased rates of reinfection in undernourished children are no longer significant when controlling for maternal literacy, income and latrine access [15], [16]. We found, however, that after accounting for poverty related factors (maternal education, HWI, latrine use) that may influence child height-for-age, children who were shorter for their age (a sign of chronic malnutrition) became more heavily reinfected and thus may be more susceptible to hookworm and *Ascaris* infection. The fact that HAZ was associated with *Ascaris* reinfection in Cycle 1 but not Cycle 2 was an intriguing finding, as was the finding that younger (not older children) had higher epgs. It is possible that the relationship between stunting and reinfection intensity is easier to detect in younger children, given that intensity of infection increases rapidly with age [51], [52], [68]. Child latrine use was also associated with a lower reinfection burden, demonstrating the importance of sanitation for reducing infection and transmission, as has been shown previously [16], [21].

In addition to individual level variables, household factors also contributed to *Ascaris* reinfection. Household risk factors commonly associated with poverty (low maternal education, low HWI) were related to increased reinfection intensity in either or both reinfection Cycles. Indeed, low maternal education [21], [25], [26] is commonly associated with greater infection burdens, likely due to poor home sanitation and hygiene practices as well as a reduced use of health services [69]. Interestingly, household poverty was associated with increased infection burden in Cycle 1 but a decreased infection intensity in Cycle 2. Examining the spatial location of infected households at the end of Cycle 2, we determined that the infections were primarily in an area of greater relative wealth. Thus it is possible that risk of infection was greater in that area and it wasn't HWI per se that influenced *Ascaris* reinfection dynamics in Cycle 2. Of further note is that although lower household poverty was associated with greater reinfection burden, other poverty related variables that may be more directly linked to transmission (latrine use, maternal education) still demonstrated the expected relationship with infection burden.

It is important to recognize that this study had a few limitations. First, we do not have data on the history of treatment prior to the baseline of Cycle 1 although we know ABZ was available in the area. Second, seasonal work-related migration reduced the number of preschool children available at the end of Cycle 1. Fortunately many children were at home three weeks later. Hence the 3 wk mean epg in Cycle 2 incorporates data from children who had just received Albendazole and children who had not been treated. Also, we were able to provide ABZ treatment at 3 wks to those who were infected but logistics prevented us from confirming the ERR following this treatment. We assume that the available ERR data can be extrapolated to all the children, and therefore that hookworm epgs were reduced significantly after the additional children were treated at 3 wks. Third, a single ABZ treatment did not remove all *Trichuris* and hookworm parasites. For the reasons noted above, we were unable to determine whether the second dose of ABZ successfully cleared infection. Fourth, we likely underestimated *Trichuris* and hookworm prevalence and intensity in Cycle 1 because we did not use the FLOTAC technique. Although this limited our ability to compare reinfection dynamics between Cycle 1 and Cycle 2, we were still able to comment on the period of most rapid transmission by verifying the trends observed with Kato Katz data for Cycle 2 (data not shown). The observed greater sensitivity of FLOTAC than duplicate Kato Katz thick smears for low intensity infections characteristic of hookworm and *Trichuris* has been recorded previously in validation studies [70]–[72] and is believed to be due to the increased volume of sample used in the FLOTAC method (1 g) compared to Kato Katz (41.7 mg). Use of both techniques in Cycle 2 alerted us that hookworm and *Trichuris* were more common in the region than previously recognized and furthermore, highlighted the low ERR for *Trichuris* after a single treatment with ABZ. In contrast to previous studies, however, Kato Katz and FLOTAC had a similar sensitivity for *Ascaris* infection. FLOTAC epg estimates were consistently lower than those calculated using Kato Katz for all 3 STH. This has also been noted previously, and could indicate that the Kato Katz technique overestimates epg due to the egg concentration that may occur while sieving the fecal sample [71], [72]. A potential consequence is the over estimation of drug efficacy through ERR when using Kato Katz. Previous work that compared ERR values between FLOTAC and Kato Katz in a drug trial [73] also found that FLOTAC estimates resulted in lower ERR values than Kato Katz. An additional contributing factor could be the ability to process diarrhetic samples using FLOTAC but not Kato Katz. However, we did not detect any difference in mean FLOTAC epg between diarrhetic vs non-diarrhetic samples. When validated these findings have implications for the monitoring of preventive control programs, in particular for the perceived success of chemotherapy as well as early detection of drug resistance. Finally, it is likely that the absence of clusters in Cycle 1 was due to the low prevalence of infection in that cycle (Hookworm: Cycle 1 = 5%, Cycle 2 = 22%; *Trichuris*: Cycle 1 = 1%, Cycle 2 = 10%) which limited our ability to detect clusters.

Our study of STH reinfection dynamics in the comarca Ngäbe Buglé of Western Panamá has emphasized that even within regions of extreme poverty, clusters of STH infections exist and that transmission is related to household level exposure variables as well as individual factors that may influence susceptibility. Our results have specific implications for public health interventions. First, the lower treatment efficacy of ABZ for *Trichuris* together with high infection levels during Cycle 2 calls attention to the importance of monitoring drug efficacy, especially against *Trichuris*, as well as the possibility of using multiple treatments or an alternative anthelmintic. Second, improving both the regional and household level sanitation and hygiene environment will be necessary to further reduce STH transmission. This will be especially important for the growth and development of stunted children who may be more susceptible to hookworm and *Ascaris* infection. Taken together, comprehensive control programs that combine short term morbidity control with the development of long term economic capacity, sanitation infrastructure and improved food security are necessary to make lasting improvements in child health in the comarca Ngäbe Buglé.
